# Design and Simulation of a 19-Electrode MEMS Piezoelectric Thin-Film Micro-Deformable Mirror for Ophthalmology

**DOI:** 10.3390/mi15040539

**Published:** 2024-04-17

**Authors:** Yisen Hu, Hongbo Yin, Maoying Li, Tianyu Bai, Liang He, Zhimin Hu, Yuanlin Xia, Zhuqing Wang

**Affiliations:** 1 School of Mechanical Engineering, Sichuan University, Chengdu 610065, China; huyisen@stu.scu.edu.cn (Y.H.); limaoying@stu.scu.edu.cn (M.L.); baitianyu@stu.scu.edu.cn (T.B.); hel20@scu.edu.cn (L.H.); 2 Key Laboratory of Radiation Physics and Technology of Ministry of Education, Institute of Nuclear Science and Technology, Sichuan University, Chengdu 610064, China; huzhimin@scu.edu.cn; 3 Med+X Center for Manufacturing, West China Hospital, Sichuan University, Chengdu 610041, China; hongboyin@163.com; 4 Department of Ophthalmology, West China Hospital, Sichuan University, Chengdu 610041, China

**Keywords:** adaptive optics, deformable mirror, piezoelectric film, aberration correction

## Abstract

This study presents a numerical simulation-based investigation of a MEMS (micro-electromechanical systems)technology-based deformable mirror employing a piezoelectric film for fundus examination in adaptive optics. Compared to the classical equal-area electrode arrangement model, we optimize the electrode array for higher-order aberrations. The optimized model centralizes electrodes around the mirror center, which realizes low-voltage driving with high-accuracy correction. The optimized models exhibited commendable correction abilities, achieving a unidirectional displacement of 5.74 μm with a driven voltage of 15 V. The voltage–displacement relationship demonstrated high linearity at 0.99. Furthermore, the deformable mirror’s influence matrix was computed, aligning with the Zernike standard surface shape of the order 1–3. To quantify aberration correction capabilities, fitting residuals for both models were calculated. The results indicate an average removal of 96.8% of aberrations to the human eye. This underscores that the optimized model outperforms the classical model in correcting high-order aberrations.

## 1. Introduction

The fundus is a human physiological feature in which the condition of blood vessels can be directly observed without surgical incision [1]. Fundus detection equipment visualizes the condition of the tissues in the eye at the cellular level, which allows a non-invasive and real-time approach to health monitoring [2]. Images taken by equipment indicate potential risks in the early stage of ophthalmic diseases, which provides eye doctors with an accurate treatment plan formulation. Due to the irregular optical pathways created by the passage of light through various ocular structures, including the cornea, crystalline lens, and vitreous body, aberrations in the wavefront images of the fundus can occur [3]. These aberrations distort the incoming wavefront, leading to imperfections in the imaging process. By employing wavefront sensing techniques, such as Hartmann–Shack wavefront sensing or Fourier domain optical coherence tomography, it is possible to analyze and quantify these aberrations [4,5]. Subsequent correction based on the acquired aberration data enables enhancement of image quality, providing a clearer and more accurate representation of the retinal structures during fundus examination. 

**Figure 1:**
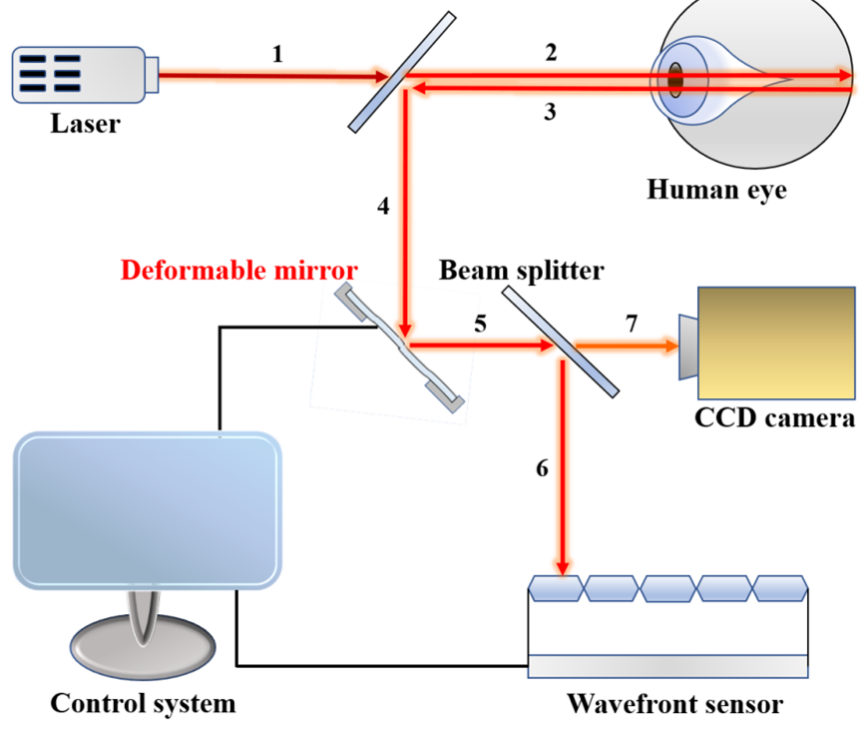
The adaptive optics system for fundus examination.

While the distortions from the optical elements can be minimized through suitable engineering, the distortions from the transmission medium, such as the atmosphere, biological tissues or the specimen itself, need active correction techniques. Conventional fundus-imaging techniques do not correct the aberration that light passes through human eye tissue. The wavefront distortion caused by the scattering of the lens and retina tissue makes the aberration effect unavoidable. Therefore, doctors have difficulty acquiring a clear fundus image. Figure 1 illustrates the proposed adaptive system, which can correct wavefront aberrations for light rays through the use of a deformable mirror and a wavefront sensor. The numbers represent the travel path of the light.

Deformable mirrors can be actuated by various mechanisms, including electrostatic actuators with cantilever designs [6,7,8,9] or magnetic actuators utilizing permanent magnets and coils [10,11,12]. These actuators induce mirror surface movement, generating displacement. In this process, electric potential is transformed into strain along the direction of material polarization, leading to displacement. Typically, a thin layer of piezoceramic material with segmented electrodes is either directly deposited or glued beneath the reflective surface to achieve unimorph bending deformation [13]. Alternatively, an array of stack actuators with thickness actuation mode may be employed for reflective surface deformation [14,15,16,17,18,19,20,21,22,23,24]. Ma et al. designed a deformable mirror using a dual-electrode piezoelectric ceramic thick film actuator [25]. However, measurements revealed that the actuator had a stroke of only 6.6 μm at 100 V, indicating that the mirror required a high voltage for minimal displacement. To address these issues, this study utilizes a piezoelectric film, aiming to achieve low power consumption with a substantial stroke.

In this study, we designed a deformable mirror utilizing a piezoelectric film to achieve a large stroke with low-voltage drive. The simulation model of the deformable mirror is based on micro-electro-mechanical systems (MEMS) theory, enabling high-resolution production at lower costs. Increasing the number of electrodes may reduce the size of each electrode on the same substrate, making it challenging to achieve the target stroke. Therefore, a large substrate is necessary to ensure both electrode size and mirror stroke. However, large-size substrates come with high costs and a low production pass rate [26]. Consequently, we designed a deformable mirror with different electrode array distributions to adapt to various types of wavefront correction capabilities, meeting the requirements of fundus examination with lower resolution.

## 2. Structural Design and Optimization of the Deformable Mirror

**Figure 2:**
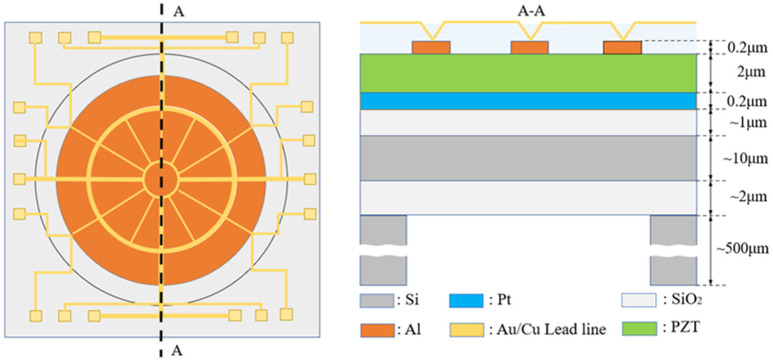
Structure design of the piezoelectric film deformable mirror.

**Figure 3:**
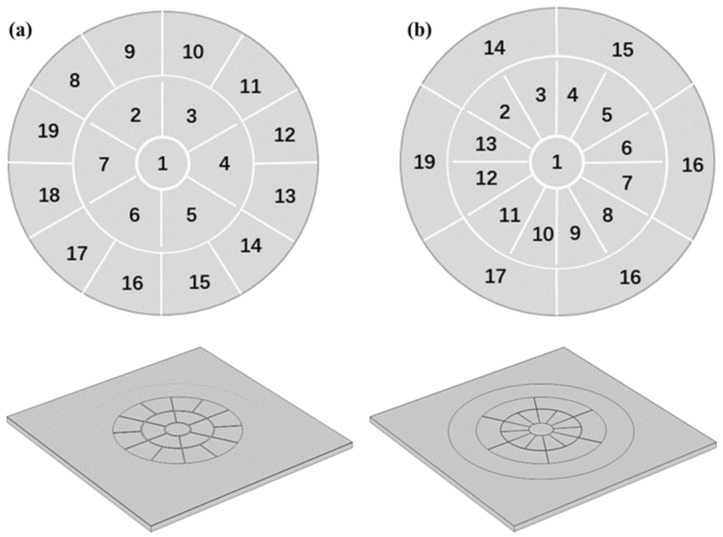
Division of top electrode array: (**a**) classical model; (**b**) optimized model.

The deformable mirror was designed to meet the requirements of fundus examination, aiming for high precision in motion with low driving voltage. Kyoto University in Japan designed and produced a piezoelectrically driven micro-mirror for adaptive optics. It is composed of a PZT thin film deposited on an silicon-on-insulator (SOI) substrate, featuring 19 electrodes distributed evenly over its surface [16]. The fabrication design of the deformable mirror is depicted in Figure 2. The model involves four layers formed on an SOI substrate (10 μm Si, 2 μm SiO_2_, and 500 μm Si). The four layers on the SOI include a 1 μm SiO_2_ bonding layer, 0.2 μm Pt lower electrode, 2 μm PZT piezoelectric layer, and 0.2 μm Al upper electrode. Additionally, there is a 15 mm diameter septum at the center serving as the movable layer of the deformable mirror. Finally, wiring is applied between the small intervals of each electrode, applying voltage to each electrode to achieve the actuation of the deformable mirror. However, this type of deformable mirror has a limited number of internal electrodes, resulting in suboptimal fitting performance. Therefore, this paper proposes a novel electrode arrangement approach. COMSOL Multiphysics 6.1 is used in the design to optimize the deformable mirror structure size [27]. The design of the deformable mirror is illustrated in Figure 2. Both types of deformable mirrors have a top electrode consisting of 19 electrodes arranged in a circular array. The classical equal-area division mode shown in Figure 3a features electrodes with equal sizes arranged sequentially along the inner and outer edges of the array. In contrast, the model shown in Figure 3b has more electrodes in the central region, with different sizes for the inner and outer electrodes. The optimized model increases the density of the inner electrode’s configuration. This design improves the resolution of the inner lens, resulting in a better ability to fit a higher-order Zernike polynomial.

In general, the size of the electrode is directly related to its kinematic capability. A higher resolution of the deformable mirror is associated with a more robust optical correction ability. Smaller-sized individual electrodes necessitate a larger operating voltage to attain the target stroke, indicating a trade-off between the kinematic capability and resolution of the deformable mirror.

In this section, the classical and optimized models of the deformable mirror were constructed using COMSOL Multiphysics 6.1 [28]. In the following sections, simulation studies were conducted to assess both the movement capability and piezoelectric properties of the PZT-5H (thin film). The dimensional parameters were then optimized based on the simulation results. Furthermore, the optical correction capability of the mirror simulation model was evaluated using the Zernike polynomial [29]. 

## 3. Optimization of Piezoelectric Thin Film Design

In the design of the deformable mirror, a piezoelectric thin film was selected as an actuator [30]. Compared with the conventional piezoelectric ceramic material, this thin film material can achieve low driving voltage with a small volume. It is not only compatible with semiconductor-integrated circuits but is also easy to combine. Considering the performance requirements and work conditions of the deformable mirror, PZT-5H was selected as the material of the piezoelectric film [31]. The thinner the piezoelectric material, the lower the required driving voltage. However, if the piezoelectric thin film is too thin, the movement of the piezoelectric materials is unable to meet the stroke standard for fundus inspection.

**Figure 4:**
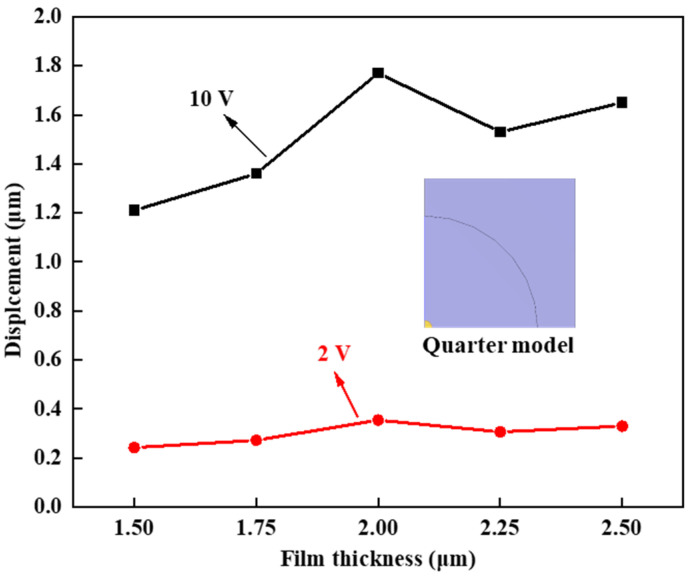
Displacement voltage diagram of piezoelectric film.

In the quarter model, the thickness of the PZT layer varied from 1.5 μm to 2.5 μm in increments of 0.25 μm. Voltages of 2 V and 10 V were applied to the yellow part with a radius of 1 mm to measure the center displacement of the mirror. As depicted in Figure 4, when the thickness of the piezoelectric film was less than 2 μm, the displacement of the center increased. This phenomenon can be attributed to the heightened thickness of the piezoelectric film, leading to an enhancement of the inverse piezoelectric effect. However, as the film thickness continued to increase, the structural constraints of the film limited its movement capacity. Considering the performance of different film thicknesses under various voltages, the optimal thickness for the piezoelectric film was determined to be 2 μm.

## 4. Optimization of Piezoelectric Thin Film Design

**Figure 5:**
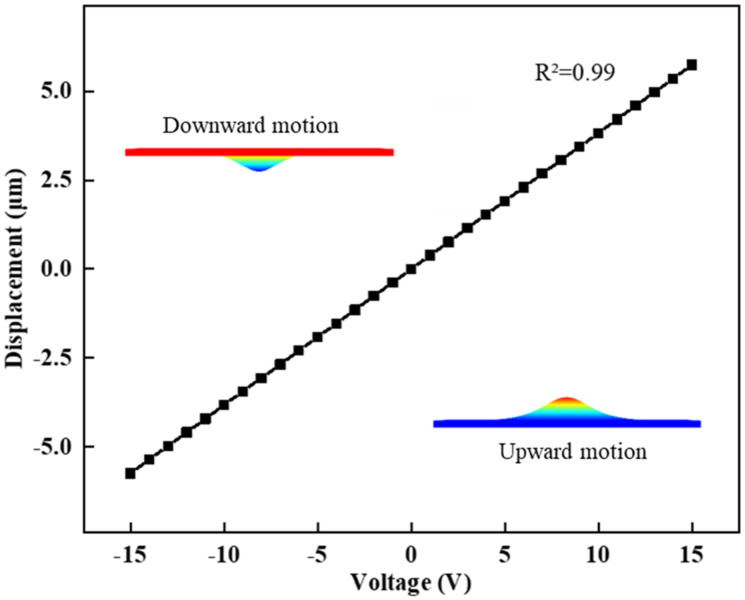
Static characteristics of the voltage displacement of the deformable mirror.

The static property of the deformable mirror was subjected to testing, wherein a voltage ranging from minus 15 V to 15 V was applied to the top electrode at 1 V intervals. The linearity between displacement and driving voltage was determined to be 0.99. As illustrated in Figure 5, the maximum displacement in the direction of the z-axis was found to be 5.74 μm, satisfying the prerequisites for fundus examination. Additionally, the maintenance of satisfactory linearity holds significant importance in accurately calculating the driving voltage for the deformable mirror.

**Figure 6:**
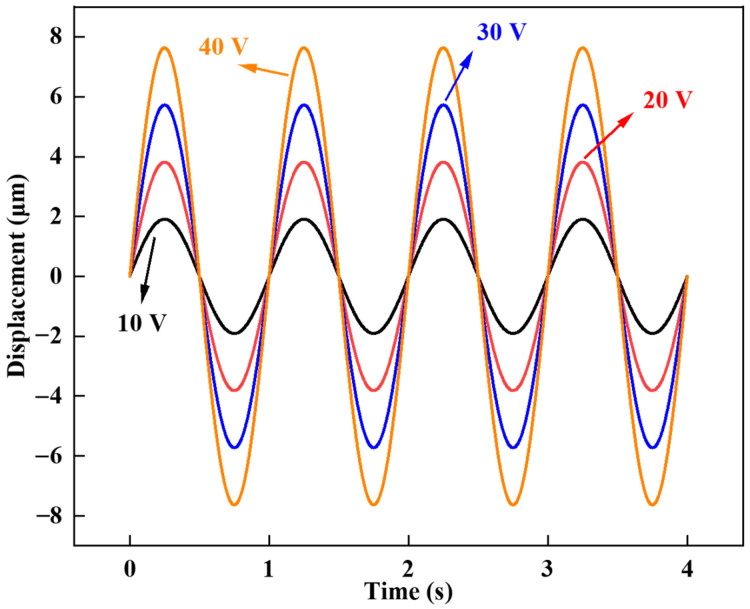
Dynamic characteristics of the voltage displacement of the deformable mirror.

The dynamic characteristics of the deformable mirror were assessed through a series of measurements. Upon applying a continuous alternating voltage to the mirror, we observed intermittent variations in displacement. Figure 6 illustrates the application of sinusoidal AC with varying peak voltages to the deformable mirror’s electrodes. 

The displacement is measured at intervals of 0.005 s over a 4-s duration. Notably, the variation trend on the mirror surface closely follows the sinusoidal curve. Furthermore, the curves exhibit stability, indicating the consistency in the relationship between displacement and driving voltage throughout the four measured periods.

## 5. Evaluation of Optical Properties of Deformable Mirror

With the objective of determining the prevalence of optical aberrations in individuals, Porter et al. conducted measurements of wavefront aberrations in the eyes of 109 healthy subjects using the Hartmann–Shack wavefront sensor [32]. Their findings revealed that the primary component of eye aberrations was the Zernike pattern of the first three orders. Consequently, this study adopts the Zernike polynomial as a criterion to assess the correction capability of the deformable mirror. Moreover, the Zernike pattern serves as a representation of the standard surface shape of the mirror.

According to the electrode array, the upper electrode is divided into multiple independent areas. Each electrode is controlled to meet the target shape. In the energizing region of the deformable mirror, voltage v_i_ is arranged in N rows, forming vector v. The type is as follows:

(1) $$v=\left(\begin{array}{c} v_{1}\\ v_{2}\\ \vdots \\ v_{N} \end{array}\right)$$

Linearity is between the applied voltage and the displacement of the movement region. The formula describing the shape of the movement region A and the applied voltage V can then be expressed. An M rows and N columns influence matrix is utilized to describe the relationship. The formula is as follows:

(2) $$\left(\begin{array}{c} a_{1}\\ a_{2}\\ \vdots \\ a_{M} \end{array}\right)=\left(\begin{array}{cccc} b_{11} & b_{12} & \cdots & b_{1N}\\ b_{21} & b_{22} & \cdots & b_{2N}\\ \vdots & \vdots & \ddots & \vdots \\ b_{M1} & b_{M2} & \cdots & b_{MN} \end{array}\right)\left(\begin{array}{c} v_{1}\\ v_{2}\\ \vdots \\ v_{N} \end{array}\right)$$

The row and column length of the influence matrix B is not the same. As a result, there is no invertible matrix. We introduce the concept of pseudo-inverse matrix B+ to replace the inverse matrix of the influence matrix [33], which consists of N rows and M columns.

Singular value decomposition of pseudo-inverse matrix B^+^ based on influence matrix B “B = ULV^T^” is shown as follows [16]:

(3) $$B^{+}={\left(ULV^{T}\right)}^{-1}={\left(V^{T}\right)}^{-1}L^{-1}U^{-1}=VL^{-1}U^{T}$$

To reproduce the shape of the movement region, the applied voltage v can be obtained by the following formula:

(4) $$v=B^{+}a$$

When the optimal voltage of the target shape a_0_ applies to the deformable mirror, the actual movement region shape a_g_ is solved as follows:

(5) $$a_{g}=BB^{+}a_{o}$$

After obtaining the control voltage to produce the Zernike polynomial of each order, the corresponding voltage is applied to each unit of the top electrode. The movement of the classical and optimized model is measured separately. The process makes use of the single numbering method. When evaluating wavefront compensation, the Z0 (piston), Z1 (x-tilt), and Z2 (y-tilt) are commonly excluded [34]. Therefore, this study chooses the first to third orders of the Zernike polynomial as fitting objects [35]. 

In this paper, we present a practical solution for correcting Zernike aberrations up to the third order by employing various combinations of electrode arrays. Given that the Tilt X, Tilt Y, and Power aberrations of the first order can be corrected using adaptive prisms, our emphasis in the aberration correction of the deformable mirror is on addressing the Astigmatism X, Astigmatism Y, Coma X, Coma Y, Trefoil X, and Trefoil Y modes of the second- and third-order Zernike coefficients.

**Figure 7:**
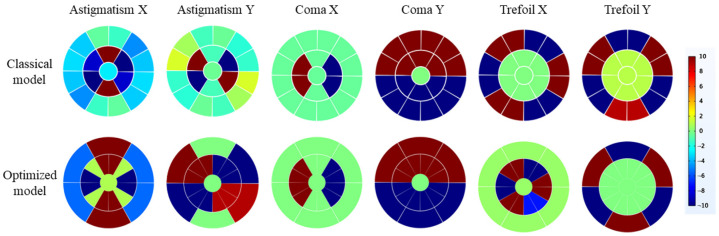
Illustration of the voltage arrays applied to the deformable mirror to achieve different deformations.

Due to the high driving voltage, the deformable mirror exhibits an unavoidable hysteresis effect, thereby affecting the kinematic accuracy of the model. The applied voltage is constrained within the range of ±10 V, as illustrated in Figure 7, corresponding to the respective driving voltage combination. In the Coma X, Coma Y, and Trefoil Y modes, the electrode array distribution applied to both the optimized model and the classical model is identical. However, in the Astigmatism X mode of the classical model, there is uneven voltage distribution in the inner electrodes, with a greater quantity of negative voltages applied compared to positive voltages. In the Astigmatism Y mode, although an equal number of positive and negative voltages are applied, the angles between the electrodes are not distributed at 90° intervals, leading to errors in the Zernike shape. Due to the higher number of inner electrodes in the optimized model, the voltage distribution in the inner electrodes is more detailed and reasonable. Positive and negative voltages can achieve a completely symmetrical distribution, resulting in better fitting performance.

For the Trefoil X mode, the electrode distribution in the optimized model is exactly the opposite of that in the classical model. In the classical model, all the voltages applied to the electrode segments are distributed on the outer electrodes of the deformable mirror. Whilst in the optimized model, all the voltages applied to the electrode segments are distributed on the inner electrodes. Although applying voltages to the inner electrodes may lead to a more accurate fitting performance, it may also result in a lower fitting deformation for the optimized model.

We extracted mirror displacement data from simulations and employed Zernike polynomials in MATLAB R2019b to closely fit an ideal wavefront that resembles the desired surface. Using the fitted wavefront, we derived 36 imaging quality metrics. In particular, we specifically extracted the first 10 terms representing Zernike polynomials of orders 1 to 3.

**Figure 8:**
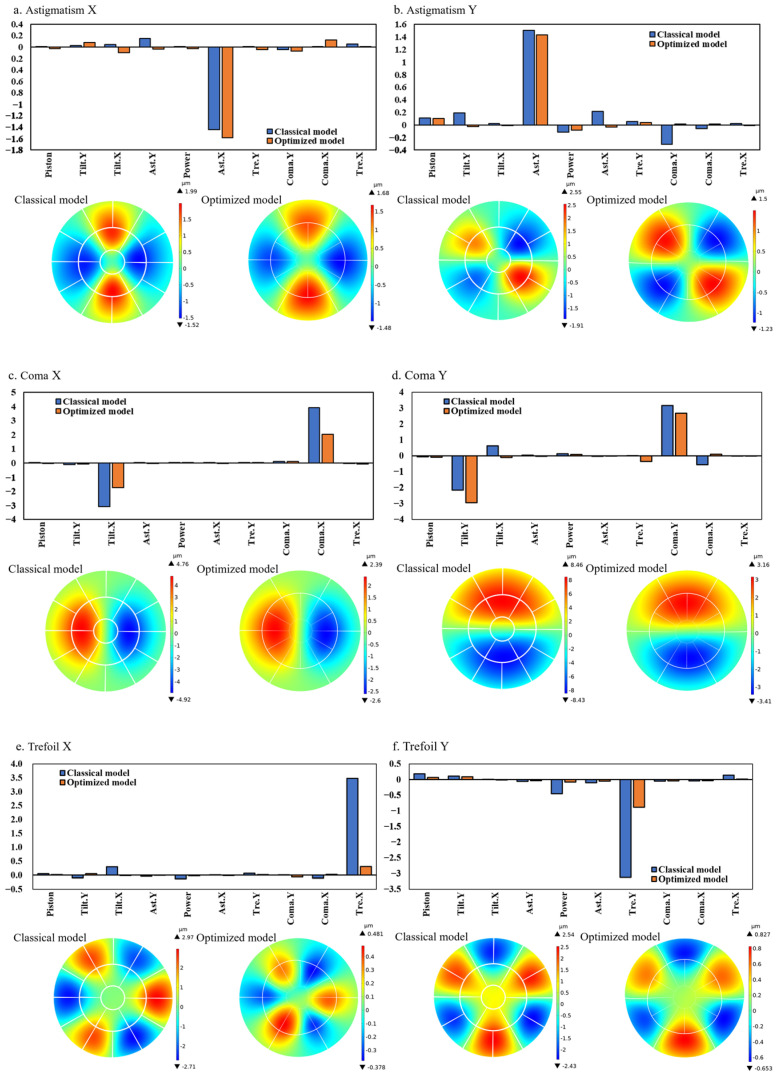
The various surface deformation displacements represented by Zernike coefficients: (**a**) Astigmatism X; (**b**) Astigmatism Y; (**c**) Coma X; (**d**) Coma Y; (**e**) Trefoil X; (**f**) Trefoil Y.

**Figure 9:**
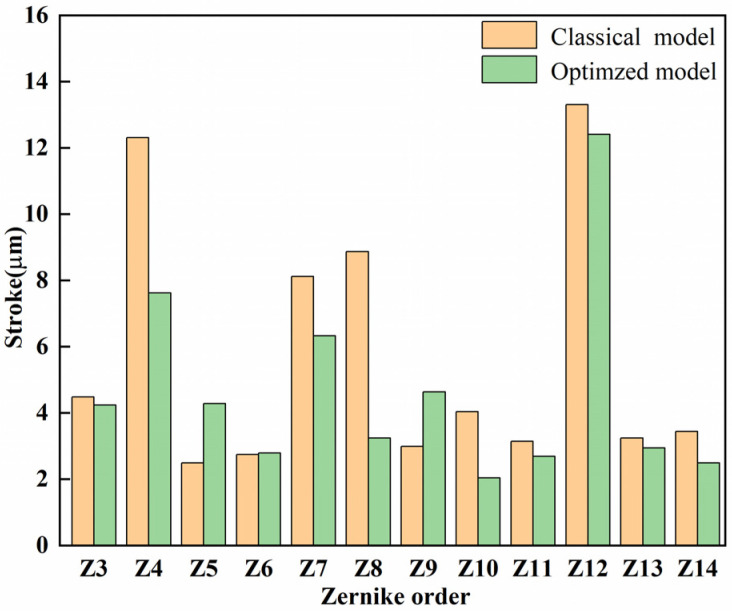
Stroke comparison of the optimized model and classical model.

The surface deformations in Figure 8 correspond to the electrode arrays depicted in Figure 9. In the correction of spherical aberrations, two deformable mirrors with different electrode arrangements have demonstrated excellent performance. However, the simulation results of the classical model indicate a more pronounced coupling phenomenon compared to other modes. For example, in the Astigmatism Y mode of the classical model, the ratio of coma Y to Astigmatism Y reaches 23.33%. The cause of this phenomenon may be the nonlinearity of piezoelectric materials or coupling introduced by the geometric shape of the electrode patches. Nevertheless, in the simulation results of the optimized model, this ratio is nearly negligible, amounting to 0.28%.

A similar issue arises in the Coma and Trefoil modes, suggesting that the optimized model exhibits better linearity, effectively reducing the coupling effects introduced by the geometric shape of the electrode patches.

Both the classical model and the optimized model demonstrate effective performance in correcting Coma aberrations. It is essential to highlight that both versions exhibit aberrations in the Tilt mode. Nevertheless, as mentioned earlier, Tilt X and Tilt Y aberrations can be corrected using adaptive prisms or digital correction techniques. Consequently, these Tilt aberrations do not exert a significant impact on the assessment of imaging quality.

In addressing the aberrations of Trefoil X and Trefoil Y modes, the performance of classical model is notably superior to optimized model. This is intuitively reflected in their corresponding Zernike coefficients, where the Trefoil X and Trefoil Y coefficients for the classical model are 3.48 and −3.12, respectively. Those for optimized model are 0.31 and −0.86. The underlying cause of this difference may be the relatively fewer external electrodes in the optimized model compared to the classical model.

For handling Trefoil X aberrations, the classical model employs external electrode driving, whereas the optimized model can only use internal electrode driving with a smaller driving area, resulting in insufficient driving force. When addressing Trefoil Y aberrations, the optimized model has only half the number of external electrodes compared to the classical model, leading to limited driving flexibility. Therefore, when dealing with images prominently featuring Trefoil aberrations, opting for the electrode arrangement of the classical model may be a preferable choice.

Figure 9 shows the stroke of two models when they fit different Zernike modes. The low-order term shapes are fitted with a greater stroke. When the order increases, the fitting displacement decreases. In addition, as the Zernike order becomes larger, the complexity of the corresponding surface augments. Due to the structure of the deformable mirror, the coupling effect exists between the electrodes. which leads to a decrease in the stroke of the deformable mirror.

The product of the pseudo-inverse and influence matrix is not an identity matrix. As a result, there are differences between the generated and standard surface shape. After calculating and comparing the residuals of two displacements, the results may indicate the difference between the two models. 

As a wavefront corrector in adaptive optics (AO) systems, the deformable mirror’s ability to fit wavefront aberrations within the fitting aperture is one of the important performance metrics. The fitting error coefficient for the i-th Zernike aberration of the deformable mirror within the fitting aperture (fixed at 10 mm to ensure that the voltage applied to each electrode does not exceed 10 V) is defined as follows [36]:

(6) $$r=\sqrt{\sum_{j=1}^{M}{\left(a_{oj}-a_{gj}\right)}^{2}}$$

**Figure 10:**
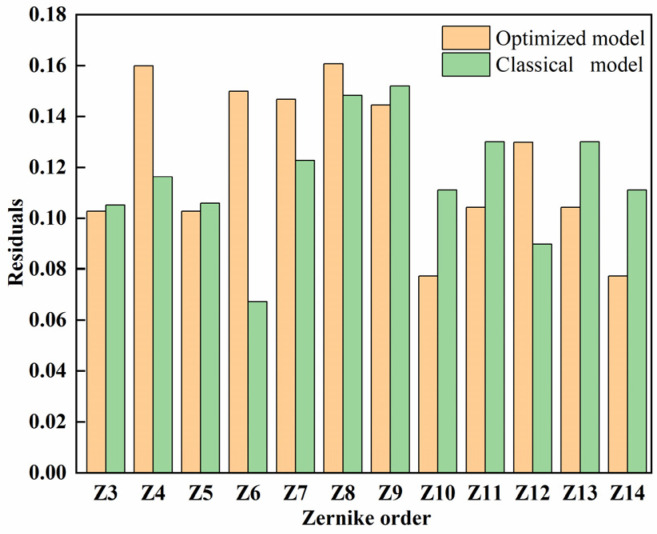
Residuals statistics between growth target shape and actual shape.

The residual calculation results are shown in Figure 10. The deformable mirrors of the two models are similar in low-order aberration correction. However, in the fourth-order (Z10–Z14) surface shape fitting, the residual error of the optimized model decreases. Meanwhile, compared with the classical model, the fourth-order surface shape residual average is 13.87% smaller.

The deformable mirror produces various strokes of different Zernike modes, resulting in a residual value between the actual and the target surface. Therefore, the result enables evaluation of the capability of aberration correction. The RMS value of eyeball wavefront aberration is j. The value between the aberration and the shape of the deformable mirror is r. Since there is no initial deviation of the deformable mirror, the removal rate of eye aberration p is as follows

(7) $$p=\frac{j-r}{j}\times 100\%$$

**Figure 11:**
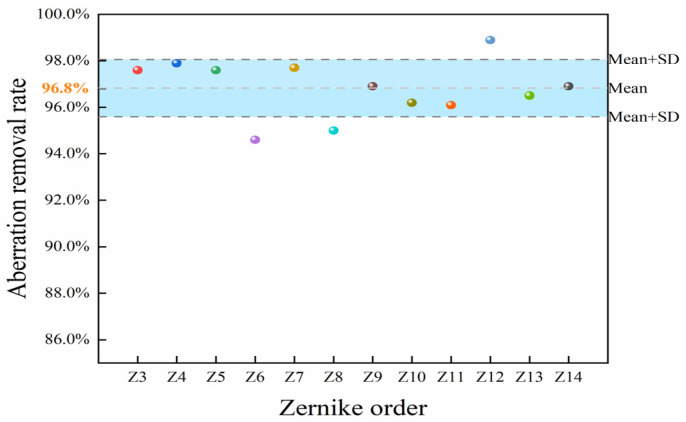
The aberration removal rate.

The calculation results are shown in Figure 11. Each colorful ball corresponds to a difference removal rate of one of the Zernike orders of the optimized model. These results are based on the optimized model, showing an average aberration removal rate of 96.8% and a maximum aberration removal rate of 98.9%.

By applying Zernike theory, devices like fundus cameras and optical coherence tomography (OCT) systems can precisely correct aberrations and improve image quality. Zernike polynomials provide a robust mathematical framework for characterizing and correcting optical aberrations in these systems. This correction is crucial for high-resolution visualization of the retina, aiding accurate diagnosis and monitoring of retinal diseases. Adaptive optics technology using Zernike deformable mirrors dynamically corrects aberrations induced by the eye’s optics in real time, enhancing imaging resolution and contrast. Thus, Zernike theory advances fundus cameras and OCT systems, enabling early detection and personalized management of retinal pathologies.

## 6. Conclusions

In this study, a piezoelectric deformation mirror employing MEMS technology for a fundus examination adaptive optics system was designed. The model was optimized and evaluated using COMSOL Multiphysics 6.1 The selected material of the actuator was PZT-5H, offering a small size and low-voltage driving.

Compared to the classical equal-area electrode arrangement model, we optimized an electrode array for higher-order aberrations. The optimized model centralized electrodes around the mirror center, which realized low-voltage driving with high-precision correction. We evaluate the kinematic performance of the deformable mirror. The optimized models exhibited commendable correction abilities, achieving a unidirectional displacement of 5.74 μm with a driven voltage of 15 V. The voltage–displacement relationship demonstrated high linearity at 0.99. In addition, the dynamic characteristics test showed that the variation tendency on the mirror surface synchronized with the voltage.

Comparing the optimized model with the classical model, Zernike polynomial fitting was employed to evaluate the correction effect of mirror aberrations. It was found that the optimized model had a closer fitting effect to the standard surface. To quantify aberration correction capabilities, fitting residuals for both models were calculated. The results indicated an average removal of 96.8% of aberrations to the human eye. In addition, the RMS was employed to evaluate the fitting accuracy. Our results showed that the optimized model fitted the third-order Zernike shape more effectively.

In general, measurements show that deformable mirrors correct optical aberration with high accuracy. As a result, this research provides significant value in the field of fundus examination and adaptive optics.

## Data Availability

The original contributions presented in the study are included in the article, further inquiries can be directed to the corresponding authors.

## References

[1] Bonora S., Jian Y., Zhang P., Zam A., Pugh E.N., Zawadzki R.J., Sarunic M.V. (2015). Wavefront correction and high-resolution in vivo OCT imaging with an objective integrated multi-actuator adaptive lens. Opt. Express.

[2] Roorda A., Romero-Borja F., Donnelly Iii W., Queener H., Hebert T., Campbell M. (2002). Adaptive optics scanning laser ophthalmoscopy. Opt. Express.

[3] Lombardo M., Serrao S., Devaney N., Parravano M., Lombardo G. (2013). Adaptive Optics Technology for High-Resolution Retinal Imaging. Sensors.

[4] Vacalebre M., Frison R., Corsaro C., Neri F., Conoci S., Anastasi E., Curatolo M.C., Fazio E. (2022). Advanced Optical Wavefront Technologies to Improve Patient Quality of Vision and Meet Clinical Requests. Polymers.

[5] Kanngiesser J., Roth B. (2020). Wavefront Shaping Concepts for Application in Optical Coherence Tomography—A Review. Sensors.

[6] Bifano T.G., Krishnamoorthy Mali R., Dorton J.K., Perreault J., Vandelli N., Horenstein M.N., Castanon D.A. (1997). Continuous-membrane surface-micromachined silicon deformable mirror. Opt. Eng..

[7] Bush K., German D., Klemme B., Marrs A., Schoen M. (2004). Electrostatic Membrane Deformable Mirror Wavefront Control Systems: Design and Analysis.

[8] Bifano T., Bierden P., Perreault J. (2004). Micromachined Deformable Mirrors for Dynamic Wavefront Control.

[9] Bonora S., Poletto L. (2006). Push-pull membrane mirrors for adaptive optics. Opt. Express.

[10] Cugat O., Basrour S., Divoux C., Mounaix P., Reyne G. (2001). Deformable magnetic mirror for adaptive optics: Technological aspects. Sens. Actuators A Phys..

[11] Brousseau D., Borra E.F., Thibault S. (2007). Wavefront correction with a 37-actuator ferrofluid deformable mirror. Opt. Express.

[12] Hamelinck R., Ellenbroek R., Rosielle N., Steinbuch M., Verhaegen M., Doelman N. (2008). Validation of a New Adaptive Deformable Mirror Concept.

[13] Gowda H.G.B., Wallrabe U., Wapler M.C. (2023). Higher order wavefront correction and axial scanning in a single fast and compact piezo-driven adaptive lens. Opt. Express.

[14] Sato T., Ishida H., Ikeda O. (1980). Adaptive PVDF piezoelectric deformable mirror system. Appl. Opt..

[15] Hishinuma Y., Yang E.-H. (2006). Piezoelectric unimorph microactuator arrays for single-crystal silicon continuous-membrane deformable mirror. J. Microelectromech. Syst..

[16] Kanno I., Kunisawa T., Suzuki T., Kotera H. (2007). Development of deformable mirror composed of piezoelectric thin films for adaptive optics. IEEE J. Sel. Top. Quantum Electron..

[17] Yang P., Liu Y., Yang W., Ao M.-W., Hu S.-J., Xu B., Jiang W.-H. (2007). Adaptive mode optimization of a continuous-wave solid-state laser using an intracavity piezoelectric deformable mirror. Opt. Commun..

[18] Norton A., Evans J., Gavel D., Dillon D., Palmer D., Macintosh B., Morzinski K., Cornelissen S. (2009). Preliminary Characterization of Boston Micromachines’ 4096-Actuator Deformable Mirror.

[19] Verpoort S., Wittrock U. (2010). Actuator patterns for unimorph and bimorph deformable mirrors. Appl. Opt..

[20] Wlodarczyk K.L., Bryce E., Schwartz N., Strachan M., Hutson D., Maier R.R., Atkinson D., Beard S., Baillie T., Parr-Burman P. (2014). Scalable stacked array piezoelectric deformable mirror for astronomy and laser processing applications. Rev. Sci. Instrum..

[21] Wang H. (2017). Research on a bimorph piezoelectric deformable mirror for adaptive optics in optical telescope. Opt. Express.

[22] Zhu Z., Li Y., Chen J., Ma J., Chu J. (2017). Development of a unimorph deformable mirror with water cooling. Opt. Express.

[23] Toporovsky V., Kudryashov A., Skvortsov A., Rukosuev A., Samarkin V., Galaktionov I. (2022). State-of-the-Art Technologies in Piezoelectric Deformable Mirror Design. Photonics.

[24] Han X., Ma J., Bao K., Cui Y., Chu J. (2023). Piezoelectric deformable mirror driven by unimorph actuator arrays on multi-spatial layers. Opt. Express.

[25] Ma J., Li B., Zhang J.H., Xu X., Chu J. (2012). PZT-thick-film actuators driven by double electrodes for MEMS deformable mirror. Nami Jishu Yu Jingmi Gongcheng/Nanotechnol. Precis. Eng..

[26] Tsuda S., Suzuki T., Kanno I., Kotera H. High-resolution Piezoelectric Deformable Mirror for Adaptive Optics. Proceedings of the Conference on Information, Intelligence and Precision Equipment.

[27] Yi W. (2022). Deformable Reflection Micromirror with Piezoelectric Actuation Based on MEMS Process. Micronanoelectron. Technol..

[28] Zhang Z., Wu Z.-Z., Jiang X.-X., Wang Y.-Y., Zhu J.-L., Li F. (2018). Modeling and experimental verification of surface dynamics of magnetic fluid deformable mirror. Acta Phys. Sin..

[29] Zhu L., Sun P.C., Bartsch D.U., Freeman W.R., Fainman Y. (1999). Wave-front generation of Zernike polynomial modes with a micromachined membrane deformable mirror. Appl. Opt..

[30] Dong W., Lu X., Cui Y., Wang J., Liu M. (2007). Fabrication and characterization of microcantilever integrated with PZT thin film sensor and actuator. Thin Solid. Films.

[31] Chaplya P., Carman G. (2002). Compression of PZT-5H Piezoelectric Ceramic at Constant Electric Field: Investigation of Energy Absorption Mechanism.

[32] Porter J., Guirao A., Cox I.G., Williams D.R. (2001). Monochromatic aberrations of the human eye in a large population. J. Opt. Soc. Am. A Opt. Image Sci. Vis..

[33] Fernández E.J., Artal P. (2003). Membrane deformable mirror for adaptive optics: Performance limits in visual optics. Opt. Express.

[34] Li J., Chen X. (2013). Aberration compensation of laser mode unging a novel intra-cavity adaptive optical system. Optik.

[35] Roddier F., Northcott M., Graves J.E. (1991). A simple low-order adaptive optics system for near-infrared applications. Publ. Astron. Soc. Pac..

[36] Chung S.-W., Shin J.-W., Kim Y.-K., Han B.-S. (1996). Design and fabrication of micromirror supported by electroplated nickel posts. Sens. Actuators A Phys..

